# Measures of Referral vs Receipt of Social Services Among Patients With Health-Related Social Needs

**DOI:** 10.1001/jamanetworkopen.2024.7021

**Published:** 2024-04-17

**Authors:** Fred S. Johnson, Eugenia R. McPeek Hinz, David Regan, Raman Nohria, Gene Moon, Susan E. Spratt

**Affiliations:** 1 Department of Family Medicine and Community Health, Duke School of Medicine, Durham, North Carolina; 2 Duke Population Health Management Office, Durham, North Carolina; 3 Duke Health Technology Solutions, Durham, North Carolina; 4 Division of General Internal Medicine, Department of Medicine, Duke School of Medicine, Durham, North Carolina; 5 Duke School of Medicine, Durham, North Carolina; 6 Division of Endocrinology, Nutrition, and Metabolism, Department of Medicine, Duke School of Medicine, Durham, North Carolina

## Abstract

This cohort study compares measures of referral vs receipt in evaluating social resource platform outcomes among patients with health-related social needs.

## Introduction

Unmet health-related social needs (HRSNs) are associated with adverse patient health outcomes, contributing to health disparities.^1,2,3,4,5,6^ On January 1, 2024, the Centers for Medicare & Medicaid Services required hospitals to screen patients for 5 HRSN domains: food, transportation, housing, violence, and utilities. An expected consequence was finding more patients with HRSNs, necessitating a process to ensure that they were connected to resources, which is often complicated with multiple steps and points of failure.^1^ Duke Health has used NCCARE360/Unite Us, the first statewide social resource connection platform^6^ embedded in our electronic health record to place and track referrals.

To monitor successful use, the platform uses the closed loop rate (CLR), or the number of patient cases that are marked as closed divided by the total number of cases. Cases are marked as closed irrespective of whether patients receive a resource. We developed a patient-centered metric, successful connection rate (SCR), to define real benefit by excluding vague outcomes, such as resource lists or general information. We conducted a retrospective cohort analysis to compare CLR and SCR.

## Methods

This cohort study is reported following the STROBE reporting guideline. Our work to review social determinants of health screening, connection, and outcomes was exempt from review and consent according to the policies of the Duke University institutional review board because it was deemed a minimal risk study due to using secondary deidentified data for analysis. We pulled deidentified data, including race and ethnicity (eMethods in Supplement 1), service domain, and case outcome, from the resource connection platform Tableau dashboard for 115 316 social referrals, resulting in 83 365 managed cases across 26 counties during 2 periods with different funding for resources and personnel: period 1 (October 1, 2020, to March 1, 2021) and period 2 (October 1, 2021, to March 1, 2022). During period 1, millions of dollars in federal funding for North Carolina’s Social Support Program supported community-based organization (CBO) use of NCCARE360,^5^ funding a network of community health workers and CBOs coordinated by a lead entity. Period 2 did not provide a requirement to use NCCARE360, a closed network of community health workers and CBOs, or funding for services (aside from emergency food).

**Figure 1.**
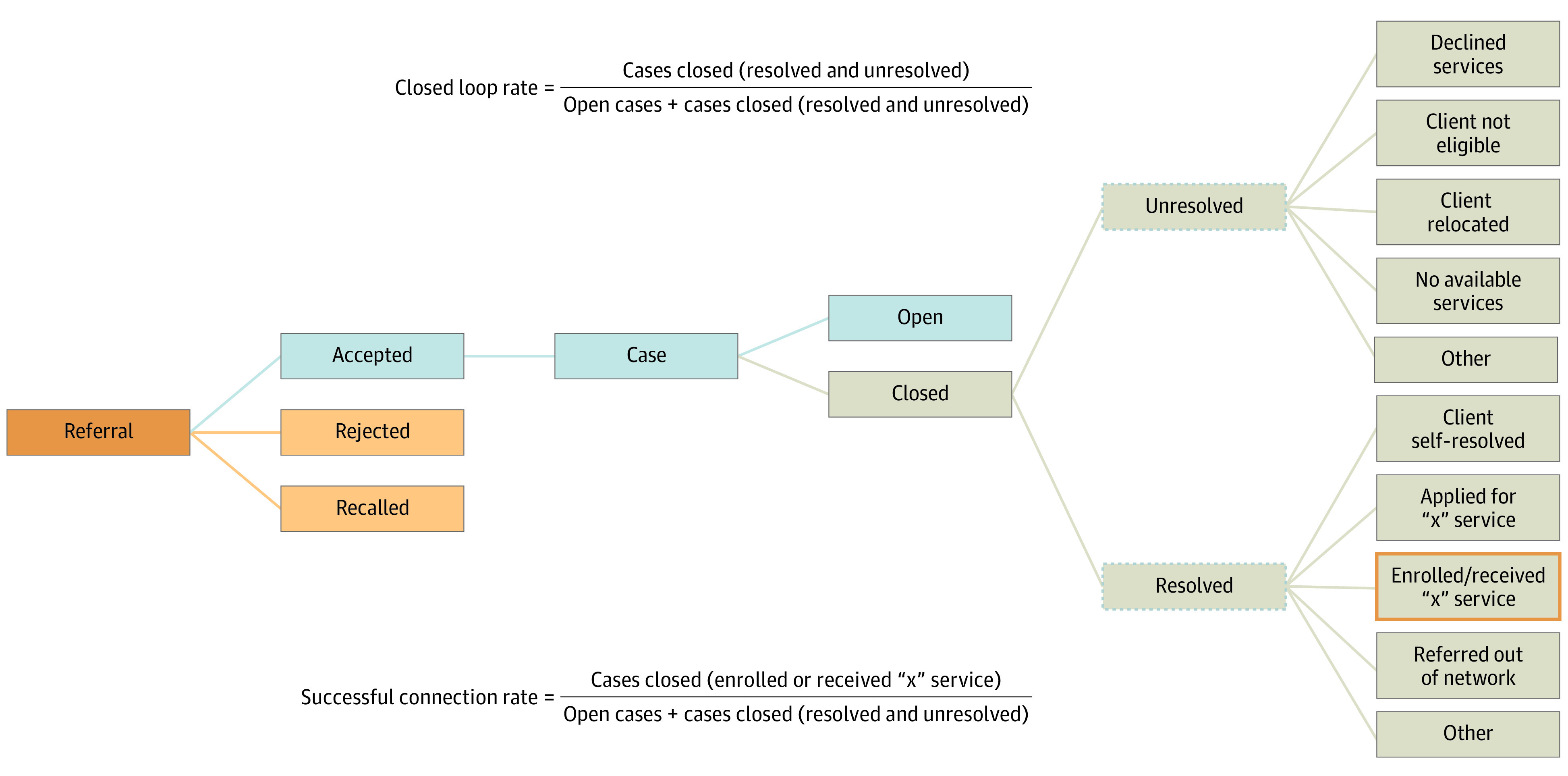
Schematic for Platform Outcomes From Referral to Case to Client Formulas for closed loop rate and successful connection rate are given.

We defined SCR as the ratio of cases whose closure resulted from receipt of real benefit divided by all managed cases (Figure 1). We used descriptive statistics to assess CLR and SCR for food, housing, utilities, and income during periods 1 and 2.

## Results

**Figure 2.**
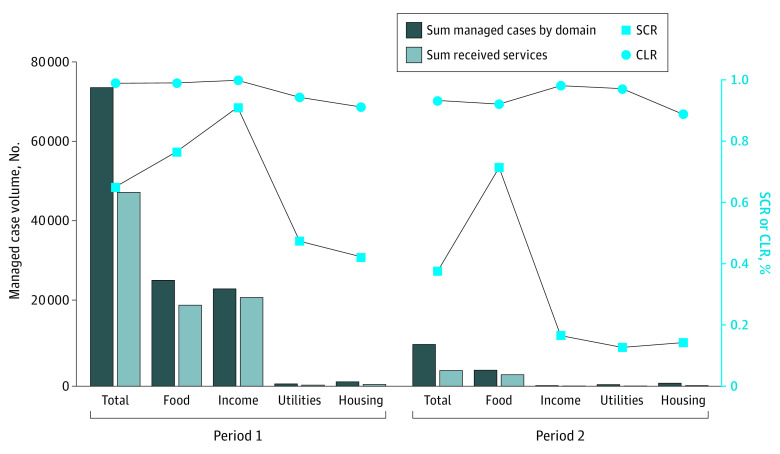
Closed Loop Rate (CLR) and Successful Connection Rate (SCR) by Period CLR vs SCR is given by volume of managed cases for period 1 (October 1, 2020, to March 1, 2021) and period 2 (October 1, 2021, to March 1, 2022).

There were 46 240 patients in period 1 (59% women, 22% men, and 18% undisclosed gender; 60% Black, 6% White, and 27% undisclosed race; 7% Hispanic) and 9529 patients in period 2 (63% women, 25% men, and 12% undisclosed gender; 54% Black, 9% Hispanic, 11% White, and 28% undisclosed race and ethnicity). We found a similar CLR in periods 1 and 2 (99% vs 93%), reflecting successful use of the NCCARE360 platform for placing and closing referrals. In contrast, we found substantial differences in the SCR between period 1 and 2 (65% vs 38%), reflecting the challenge in connecting patients to resources but also the association of funding and intermediary support for CBOs with outcomes. Notably, the SCR for the food domain (the only domain to receive continued funding) remained the same from period 1 to 2 (76% and 71%) (Figure 2).

## Discussion

NCCARE360 supports connecting patients to resources and provides data on referral outcomes. This cohort study found that CLR represented the success of the NCCARE360 platform but obscured the patient outcome. In contrast, the SCR more accurately represented whether a service was provided. The variability of the SCR was associated with funding. Adoption of social resource platforms is driven by whether patients receive services. Patient-centered metrics are essential to improvement. Successful integration of medical and social care will require financial support for resources and infrastructure. Study limitations include that the platform Tableau dashboard is a visual of dynamic data. Our findings suggest that to measure health outcomes for HRSN interventions, CLR may not be a valid representation of service connection. Although SCR came closer to measuring connection, only sustainable and meaningful changes in addressing HRSN will impact health.

## Notes

Supplement 1.
eMethods.

Supplement 2.
Data Sharing Statement

## References

[1] Schweitzer A, Pham A, Aliber M, De Kesel Lofthus A, Brooks K, Mohta NS. Applying value chain thinking to social drivers of health: a framework and two case studies. NEJM Catalyst. Published online November 17, 2021. doi:10.1056/CAT.21.0306

[2] McPeek Hinz ER, Avery C, Johnson S, Drake C, Spratt SE. Addressing health-related social needs through systematic screening and integration of a social care technology platform. NEJM Catalyst. Published online April 19, 2023. doi:10.1056/CAT.22.0324

[3] Oronce CIA, Miake-Lye IM, Begashaw MM, Booth M, Shrank WH, Shekelle PG. Interventions to address food insecurity among adults in Canada and the US: a systematic review and meta-analysis. JAMA Health Forum. 2021;2(8):e212001. doi:10.1001/jamahealthforum.2021.2001 35977189 PMC8796981

[4] Nohria R, Yu J, Tu K, . Community-based organizations’ perspectives on piloting health and social care integration in North Carolina. BMC Public Health. 2023;23(1):1914. doi:10.1186/s12889-023-16722-4 37789295 PMC10548645

[5] Bleser WK, Huber KM, Crook HL, . North Carolina’s COVID-19 support services program: lessons for health policy programs to address social needs. Milbank Memorial Fund. Accessed June 7, 2022. https://www.milbank.org/publications/north-carolinas-covid-19-support-services-program-lessons-for-health-policy-programs-to-address-social-needs/

[6] North Carolina Department of Health and Human Services. NCCARE360. Accessed December 15, 2022. https://www.ncdhhs.gov/about/department-initiatives/healthy-opportunities/nccare360

