# Supplementary material for: Measures of Referral vs Receipt of Social Services Among Patients With Health-Related Social Needs
Source: JAMA Netw Open. 2024 Apr 17;7(4):e247021. doi: 10.1001/jamanetworkopen.2024.7021 (PMC11024758; doi:10.1001/jamanetworkopen.2024.7021)
Supplement: Supplement 1. — eMethods. [file jamanetwopen-e247021-s001.pdf]

# Supplemental Online Content

Johnson FS, McPeek Hinz ER, Regan D, Nohria R, Moon G, Spratt SE. Measures of referral vs receipt of social services among patients with health-related social needs. *JAMA Netw Open*. 2024;7(4):e247021. doi:10.1001/jamanetworkopen.2024.7021

**eMethods.**

This supplemental material has been provided by the authors to give readers additional information about their work.

© 2024 Johnson FS et al. *JAMA Network Open.*

**eMethods.**

Race and ethnicity were pulled from the Tableau dashboard database. This database was fed from a variety of sources, including electronic health record data, community health worker (or other resource connection platform user) observed and entered into the platform, or self report with community health worker entry into the platform. Race categories were American Indian or Alaska Native, Asian, Black or African American, Native Hawaiian or Pacific Islander, White, other race, and undisclosed. Ethnicity categories were Hispanic or Latino, not Hispanic or Latino, and undisclosed.

Race and ethnicity are separate. Because we were pulling deidentified data we cannot connect race with ethnicity. This is a limitation to the dashboard.

Race and Ethnicity are part of the Tableau dashboard because addressing social determinants of health and health-related social needs is an integral part of improving health equity.

© 2024 Johnson FS et al. *JAMA Network Open.*
