# Supplementary material for: Measures of Referral vs Receipt of Social Services Among Patients With Health-Related Social Needs
Source: JAMA Netw Open. 2024 Apr 17;7(4):e247021. doi: 10.1001/jamanetworkopen.2024.7021 (PMC11024758; doi:10.1001/jamanetworkopen.2024.7021)
Supplement: Supplement 2. — Data Sharing Statement [file jamanetwopen-e247021-s002.pdf]

Data Sharing Statement

Johnson. Measures of Referral vs Receipt of Social Services Among Patients With Health-Related Social Needs. *JAMA Netw Open*. Published online April 17, 2024. doi:10.1001/jamanetworkopen.2024.7021

Data
Data available: Yes
Data types: Other (please specify)
Additional Information: We can provide aggregate data. It is already available in the appendix. Additional data can be viewed in the Unite Us Tableau. See link below. This will likely require access approved by Unite Us.
How to access data: https://tableau.uniteus.io/views/InsightsCenter/InsightsCenter?%3Alinktarget=_self&%3Aembed=yes&%3Atoolbar=top&%3AshowShareOptions=false#1
When available: With publication

Supporting Documents
Document types: None

Additional Information
Who can access the data: Researchers with a signed data use agreement with Duke and/or Unite Us.
Types of analyses: We provided descriptive analyses. These can be found in the results and the appendix.
Mechanisms of data availability: Signed data access agreement.
Any additional restrictions: none
